# Pulmonary Glomus Tumor

**DOI:** 10.7759/cureus.38684

**Published:** 2023-05-07

**Authors:** Sudeep Acharya, Shamsuddin Anwar, Kumar Thapa, Sakura Thapa, Michael Lau

**Affiliations:** 1 Pulmonary and Critical Care Medicine, Staten Island University Hospital, Staten Island, USA; 2 Infectious Diseases, Tufts Medical Center, Boston, USA; 3 Internal Medicine, Staten Island University Hospital, Staten Island, USA; 4 Internal Medicine, Green City Hospital, Kathmandu, NPL; 5 Internal Medicine, Donald and Barbara Zucker School of Medicine at Hofstra/Northwell, Hempstead, USA

**Keywords:** benign lung nodule, isolated lung nodule, endobronchial nodule, pulmonary glomus tumor, glomus tumor

## Abstract

Glomus tumors, which account for less than 2% of soft tissue tumors, are a rare benign soft tissue neoplasm. They originated from neuro-myo-arterial glomus tissue whose primary function is regulation of the body temperature. This tissue is commonly located in the dermis or subcutis in the subungual region; however, it can be extracutaneous such as in bones, the genitourinary tract, the gastrointestinal tract, and the respiratory tract. Histologically, a glomus tumor is made of proliferating rounded or cuboidal epithelioid cells in a meshwork of blood vessels. Although a benign growth, they can rarely show malignant features with infiltration of surrounding tissue with the rapid multiplication of cells in which case it is labeled as a malignant glomus tumor. Pulmonary glomus tumors are extremely rare and most commonly occur in middle-aged men. They are mostly asymptomatic, but a small percentage of patients may present with hemoptysis and cough if there is large airway involvement. We present an interesting case of a middle-aged man presenting with cough and occasional hemoptysis, found to have an endobronchial nodular lesion, and subsequently diagnosed with a pulmonary glomus tumor.

## Introduction

Glomus tumors are rare benign soft tissue neoplasms that originate from glomus bodies that function as temperature-regulating organs [1]. Pulmonary glomus tumors are extremely rare and most commonly occur in middle-aged men [2-4]. Glomus tumors originating from the pulmonary tract are usually asymptomatic, but rarely, patients may present with hemoptysis and cough if there is large airway involvement [5,6]. We present an interesting case of an endobronchial pulmonary glomus tumor diagnosed with bronchoscopy and treated with electrocautery snare resection and argon plasma coagulation.

This article was presented as a meeting abstract at the 2022 American College of Chest Physicians meeting on October 17, 2022.

## Case presentation

**Figure 1:**
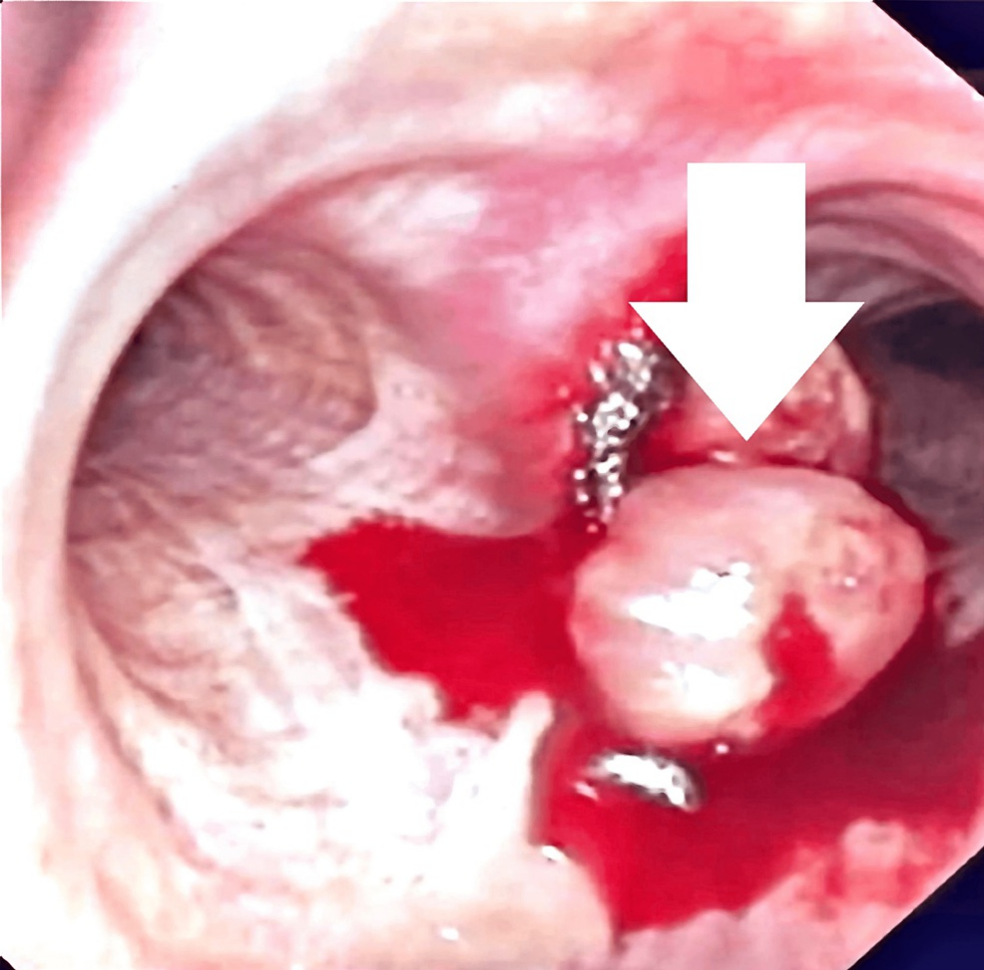
Bronchoscopic view of the right mainstem bronchus. A rounded lesion within the right mainstem bronchus was noted (arrowhead).

A 52-year-old male presented to the outpatient clinic with a chronic productive cough (yellowish sputum) and hemoptysis. He had a significant history of smoking (10 pack years) and had quit approximately 14 years before the current presentation. The computed tomography (CT) of the chest revealed a right mainstem endobronchial lesion, which was further explored with bronchoscopy, to be defined as a rounded lesion within the right mainstem bronchus (Figure 1).

The endobronchial biopsy results showed findings consistent with a glomus tumor. A positron emission tomography (PET) CT for metastasis workup was obtained, which was negative for any other metastatic foci. A repeat bronchoscopy was performed for electrocautery snare resection and argon plasma coagulation to successfully remove the tumor without complication.

## Discussion

Glomus tumors in the pulmonary tract are very uncommon findings. These are often benign growths comprising perivascular cells [7,8]. In the respiratory tract, the glomus tumors can affect the mainstem bronchi and pulmonary parenchyma. It is described as the protruding, polypoidal mass in the bronchial tract and nodular mass in the parenchymal tissue. A variety of other conditions can mimic pulmonary glomus tumors such as carcinoid tumors, leiomyoma, pulmonary sclerosing pneumocytoma, lymphoma, solitary fibrous tumor, or peripheral primitive neuroectodermal tumor [9-12]. Immunohistochemical staining is beneficial in the diagnosis of glomus tumors and in differentiating them from other conditions. Glomus tumor cells are round or oval in shape and stain-positive for type IV collagen, vimentin, and smooth muscle actin [13,14]. The mainstay of treatment of glomus tumors is surgical resection, but there is an increased utilization of endoscopic resection with successful results, as noted in our case [15]. However, it is also important to take into consideration tumor location and malignant potential in deciding between open versus endoscopic resection [16,17].

## Conclusions

Glomus tumors are benign tumors, which have been rarely described in the respiratory tract. Although these growths have very limited malignant potential, they should be investigated in pathology and imaging studies. These tumors can be managed with resection, which should be given consideration, as these vascular tumors can cause complications such as massive hemoptysis if not removed. With this case report, we intend to increase awareness among medical professionals about this rare condition. 
